# On Small Doses of Oil of Turpentine for the Cure of Sciatica and Other Neuralgiæ

**Published:** 1829-04-01

**Authors:** 


### On small Doses of Oil of Turpentine for the Cure of Sciatica and other Neuralgiæ. By M. Martinet, M.D.

The employment of terebinthinates, in affections of the nerves, is of very ancient date; but it is only in modern times that the essential oil of turpentine has been fairly put to the test of experiment in the neuralgiæ, of which sciatica is one of the most obstinate and intractable. M. Martinet has lately given an exposition of the results of his experience in 70 cases of sciatica, or other severe neuralgiæ of the extremities, where the oil of turpentine was employed, but in small doses—namely, about a fluid-drachm in the 24 hours, and taken at three different times. In this way, it seldom acted on the bowels; but generally produced a sense of heat in the stomach, and throughout the whole line of the intestinal canal, and occasional perspiration. A similar sensation was usually experienced in the nerve affected. The vehicle of exhibition was honey, syrup, or, what was the best of all, a mixture of syrup and calcined magnesia. The yolk of an egg is also a good ingredient in the vehicle. Where the digestive organs were very irritable, a few drops of laudanum were added. The modus agendi of turpentine in the cure of sciatica, or of any of the neuralgiæ, is quite unknown, and, therefore, we shall not waste time in discussing that point. The resumée of our author's experience with this remedy is as follows:—

Of seventy patients who laboured under

---

sciatica or other severe neuralgia of the extremities, fifty-eight were cured—namely, three by frictions, and fifty-five by the internal administration of the turpentine. Ten experienced only a more or less durable relief—and five derived no benefit whatever from the medicine. Two out of these last laboured under disease of the hip-joint, under which they ultimately sank. Of these 70 cases, 40 were acute, and 30 chronic. Of the 40 acute cases, 34 were cured—five were relieved—and one received no benefit. Of the 30 chronic cases, 24 were cured—two were relieved—and four remained without any mitigation of their complaints. Of the 58 patients who were completely cured, 34 were cured in less than six days—22 within twelve days—and three within six weeks. Of these 58 cures, 48 were cases of sciatica, two of which were cured by frictions. Three were crural neuralgiæ—four were brachial—and three facial. In the 10 cases which were only relieved (all of which were cases of sciatica) the treatment was suspended after the second day. In 21 cases, heat was felt along the tract of the nerve and in the limb affected; and 19 of these were completely cured. In 18 cases, heat was felt in the stomach and bowels. Three were affected with vomiting—but these had taken a larger dose than usual of the oil. Three had diarrhœa and colic. In five instances, the urine was augmented—four had dysury or strangury. In ten instances the perspiration was augmented. In one case, a degree of intoxication was produced—and in two others, a pruritus was experienced over the body.—Revue Medicale.

---

